# Integration of finite element method and artificial intelligence for evaluating PEEK composites in rib cage reconstruction process under impact conditions

**DOI:** 10.1007/s10856-025-06972-6

**Published:** 2025-12-24

**Authors:** Yomna H. Shash, Rana Hossam Elden

**Affiliations:** Biomedical Engineering Department, Faculty of Engineering, Helwan University, Cairo, Egypt

## Abstract

Rib cage reconstruction is critical for maintaining chest rigidity, protecting intrathoracic organs, and preserving vital physiological functions. Although titanium has traditionally been used for reconstruction due to its mechanical strength and biocompatibility, its limitations have prompted the search for alternative materials. The finite element method (FEM) is widely used to assess implant performance through stress analysis, while advances in artificial intelligence (AI) now allow the integration of FEM with predictive modeling to efficiently estimate mechanical responses. This study aimed to evaluate the feasibility of using PEEK and PEEK composites as alternatives to metallic implants for rib reconstruction and to develop AI models capable of predicting stresses, strains, and deformations. Customized 3D models of a defective chest were reconstructed with implants made from PEEK, carbon fiber-reinforced PEEK (CFP), glass fiber-reinforced PEEK (GFP), and hydroxyapatite PEEK (HAP) as alternatives to titanium. FEM simulations were performed under lateral impact and sternal forces to extract mechanical responses, generating a comprehensive dataset used to train machine learning and deep learning regression models, including Linear Regression, Ridge Regression, Support Vector Regression, Decision Trees, Neural Networks, and LightGBM. Model performance was evaluated using R², MAE, MSE, RMSE, and computational efficiency. Results indicated that CFP 60% implants produced the lowest stress and strain levels on ribs and lungs, whereas pure PEEK and HAP 30% implants exhibited higher levels. GFP 30% and HAP 60% implants distributed tensile and compressive stresses similarly, though HAP 60% implants were prone to fracture due to excessive tensile stresses. AI models trained on FEM data achieved over 99.9% accuracy, demonstrating both predictive reliability and computational efficiency. These findings suggest that CFP (30% & 60%) and GFP (30% & 60%) composites are promising alternatives to titanium for rib reconstruction, and that integrating FEM with AI-based regression models can significantly optimize implant evaluation and design.

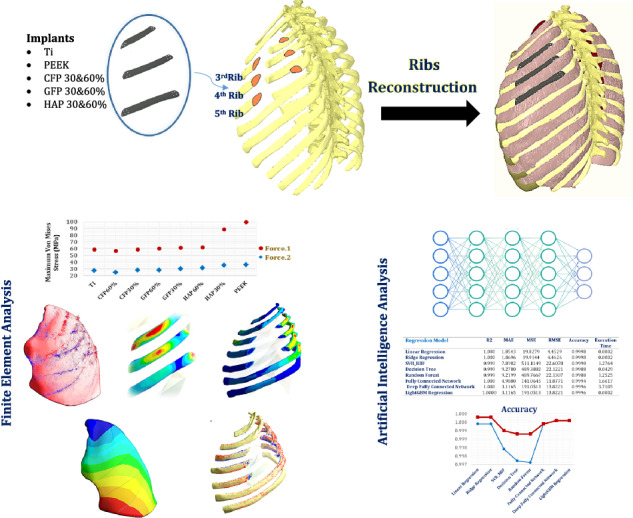

## Introduction

The bony framework of the thoracic cage consists of the ribs, sternum, and thoracic vertebrae. Chest wall defects most commonly result from surgical resection of tumors, locally invasive malignancies, metastatic lesions, or infectious processes caused by bacterial or viral agents [1, 2]. Additionally, thoracic trauma resulting from motor vehicle collisions represents the second most frequent cause of injury-related mortality after head trauma [3]. The primary objectives of rib cage reconstruction are to restore thoracic rigidity and structural stability, thereby protecting intrathoracic organs and preserving normal respiratory mechanics.

The ribs represent the most frequently damaged bones of the thoracic cage, often necessitating surgical fixation or reconstruction using plates and implants [4]. The development of rib implants has therefore become a critical aspect of thoracic reconstruction to restore chest wall integrity and mechanical durability [2]. Commonly employed materials for implant fabrication include stainless steel, cobalt–chromium alloys, commercially pure titanium, and titanium alloys [5–7]. Among these, titanium alloy (Grade 5, Ti-6Al-4V) remains the most widely used material in rib cage reconstruction process due to its favorable balance of strength, biocompatibility, and corrosion resistance [5].

Metallic implants offer several advantages, such as high mechanical strength, long-term stability, and excellent chemical and biological compatibility [8, 9]. However, their use is associated with certain drawbacks, including the need for surface modifications, manufacturing complexity, and incompatibility with imaging modalities such as CT and MRI, which often generate radiologic artifacts [10]. In addition, metal implants are prone to corrosion and wear, potentially resulting in the release of metallic ions and debris into surrounding tissues [11]. These effects can trigger hypersensitivity reactions manifested as erythema, urticaria, eczema, edema, discomfort, or even necrosis [12]. Moreover, metallic implants have been linked to secondary complications, including microbial contamination and surface degradation associated with peri-implantitis [13]. Consequently, the search for alternative biomaterials—such as advanced ceramics and high-performance polymers—has gained increasing attention for rib reconstruction applications.

When selecting materials for rib cage implants, several critical criteria must be met, including biocompatibility and adequate mechanical properties to withstand the physiological stresses associated with respiration and thoracic motion [14]. Additional factors influencing material selection include cost-effectiveness, ease of manufacturing, rapid preparation, and readiness for clinical implementation [15]. In recent years, polymeric materials have attracted considerable attention for use in bone and thoracic reconstruction due to their favorable biomechanical and biological characteristics [15–17]. Various polymers, such as polytetrafluoroethylene (PTFE), polypropylene (PP), and polymethyl methacrylate (PMMA) and its composites, have been employed in the development of rib prostheses [18].

Polyether ether ketone (PEEK) is a high-performance thermoplastic polymer increasingly utilized in orthopedic and craniofacial applications for bone fixation and reconstruction, including the fabrication of implants, plates, and fixation screws [19–21]. PEEK exhibits excellent mechanical, chemical, thermal, and electrical properties, in addition to radiolucency and proven biocompatibility in both in vivo and in vitro environments [19]. Unlike metallic materials, PEEK does not induce adverse biological reactions or imaging artifacts. Its relatively low elastic modulus and superior shock-absorbing capacity contribute to reducing stress shielding and mitigating stress concentrations in adjacent bone and soft tissues [20].

The mechanical performance of PEEK can be further enhanced by incorporating reinforcing fillers such as bioceramics, glass fibers, or carbon fibers in specific proportions [22, 23]. Pure PEEK possesses an elastic modulus of approximately 3.5 GPa; however, when reinforced with 30–60% carbon or glass fibers, the modulus can be tailored within a broad range (12–150 GPa) to better match the stiffness of cortical bone [22]. These PEEK composites combine low plaque affinity, high chemical inertness, strength, biocompatibility, and compatibility with medical imaging modalities, as they produce minimal radiologic artifacts [22, 24]. Furthermore, compared to metallic and ceramic materials, PEEK-based composites are more cost-effective and easier to process and fabricate using additive manufacturing or injection molding techniques [25].

Synthetic hydroxyapatite (HA) is a bioactive calcium phosphate ceramic widely used in dental and orthopedic applications due to its ability to chemically bond with bone tissue and promote osteointegration through surface-mediated bone growth [26]. Reinforcing PEEK with hydroxyapatite particles not only improves its mechanical properties but also enhances its bioactivity, resulting in composites that combine high stiffness with superior osseointegration potential. Hydroxyapatite-reinforced PEEK (HAP) composites exhibit excellent biocompatibility, chemical stability, and resistance to biological degradation [27]. However, their relatively low tensile strength and increased brittleness compared to unfilled or fiber-reinforced PEEK limit their use in load-bearing applications. Consequently, additional optimization and mechanical evaluation are required before HAP composites can be considered suitable for demanding applications such as chest wall reconstruction [28].

The finite element method (FEM), a computer-aided engineering (CAE) tool, is utilized to solve biomedical problems and assess the performance of designs in real-world scenarios [29]. It is particularly effective in researching biomechanical characteristics and predicting the body’s response to various mechanical configurations. FEM is especially valuable in procedure planning, implant and tool design, and creating anatomically accurate prostheses in fields such as maxillofacial surgery and bone reconstruction [30]. In chest wall reconstruction, FEM can accurately model complex geometries, address flaws, propose alternative designs, simulate different materials in various scenarios, and analyse internal stresses and strains at any point in the process [6, 31–33].

Recent advancements in artificial intelligence (AI) have revolutionized approaches to material stress analysis and biomedical engineering applications. AI-driven predictive models have emerged as powerful tools for accelerating material characterization, optimizing implant design, and enhancing decision-making in both clinical and engineering contexts. Unlike conventional finite element simulations that demand substantial computational resources, these models enable rapid estimation of mechanical responses—such as stresses, strains, and deformations—with improved efficiency and accuracy. The strength of predictive modeling lies in its ability to process and analyze extensive datasets generated from finite element simulations (FEM), allowing for precise prediction of material behavior under diverse loading conditions without the need for repetitive or time-intensive computations. Machine learning algorithms such as Support Vector Regression (SVR), Random Forests, and advanced gradient boosting frameworks like LightGBM have demonstrated strong capability in predicting mechanical properties with high accuracy, thereby reducing reliance on costly experimental testing and prolonged numerical analyses [22, 23, 31].

Several recent studies have demonstrated the potential of integrating finite element modeling (FEM) with artificial intelligence (AI)-based prediction models to transform material design and biomechanical analysis. For instance, Zhang et al. utilized FEM-generated datasets to train machine learning algorithms for predicting the mechanical responses of rib implants, achieving substantial reductions in computational time without sacrificing accuracy [31]. Similarly, Rahmitasari et al. applied AI techniques to optimize the design of PEEK-based dental implants, demonstrating the capability of prediction models to efficiently assess material performance under dynamic loading conditions [23]. Kang et al. further emphasized the role of AI in the customized design of rib prostheses, reporting improvements in patient-specific outcomes and reductions in development costs [34]. Moreover, Risteska et al. highlighted the significance of carbon fiber-reinforced PEEK composites in enhancing the mechanical and functional performance of orthopedic implants [35]. Beyond FEM-based simulations, recent research underscores the growing potential of AI to predict complex material behaviors—including wear resistance, fatigue life, and long-term performance under cyclic loading—thereby broadening its applicability in biomedical material evaluation and implant optimization [36].

This study presents a novel integration of Finite Element Method (FEM) and Artificial Intelligence (AI) to evaluate PEEK-based composite rib implants under impact conditions. By combining FEM simulations with artificial intelligence models, the approach can accurately predict stresses, strains, and deformations while significantly reducing computational time. This study also provides the first comprehensive assessment of PEEK composites—carbon fiber-, glass fiber-, and hydroxyapatite-reinforced PEEK—for rib reconstruction, identifying optimal alternatives to titanium. Hence, the study offers new insights into stress transfer mechanisms in rib–implant systems, establishing an efficient FEM–AI framework for designing next-generation thoracic implants.

The first phase of this study aims to evaluate the feasibility of using PEEK and its composite variants as alternatives to metallic implants for reconstructing resected portions of the left third, fourth, and fifth ribs, and to assess their mechanical performance using finite element analysis (FEM). Composite materials are selected for the implants because each rib can be considered a composite structure, consisting of an outer cortical layer and an inner cancellous core. FEM simulations are conducted to determine the stresses, strains, and deformations of the cortical and cancellous bone, as well as the mechanical stresses on the implants, following rib reconstruction using PEEK and various PEEK composites, including hydroxyapatite-reinforced PEEK (30% and 60%), carbon fiber-reinforced PEEK (30% and 60%), and glass fiber-reinforced PEEK (30% and 60%). Two loading scenarios are simulated: (1) lateral impact forces representative of motor vehicle collisions, and (2) sternal loading forces typical of cardiopulmonary resuscitation maneuvers.

**Fig. 1:**
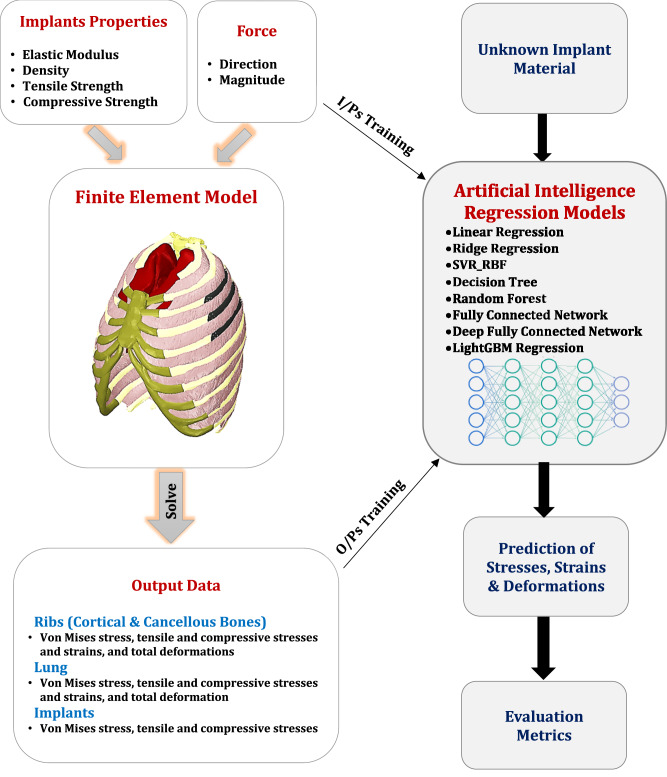
Workflow illustrating the integration of finite element modeling and artificial intelligence techniques. The framework encompasses the generation of simulation data using FEM, followed by the development of AI models for predicting stresses, strains, and deformations after rib cage reconstruction process

In the second phase of this study, the FEM is employed to generate an extensive dataset encompassing implant material properties, loading conditions, and the resulting mechanical responses. This dataset serves as the foundation for training advanced AI models designed to predict the stresses, strains, and deformations experienced by ribs and lungs when using any PEEK composite implant. By leveraging these predictive models, the need for repetitive and computationally intensive FEM simulations is significantly reduced, streamlining the evaluation process and enabling efficient material optimization, as depicted in Fig. 1.

## Materials and methods

This section was divided into two main parts: the construction of the finite element model, and the design of the artificial intelligence models

### Finite element model design

The finite element model was constructed and analysed by the following steps:

#### Model generation and rib cage reconstruction

The detailed three-dimensional model of healthy human chest, including the sternum, 24 ribs, cartilages, 12 thoracic vertebrae, 2 lungs, and the internal, external, and innermost intercostal muscles, was downloaded as OBJ files from the anatomical database “BodyParts3D/Anatomography” (BodyParts3D, Life Sciences Integrated Database Center, Japan) [37]. These files were based on computerized tomography (CT) images of a 22-year-old volunteer with a body mass index of 21.7 who underwent CT scans every 2 mm from the top of the head to the feet in a standing position [38].

The OBJ files of the human chest were then exported to the “Space Claim” program for improvement, adjustment, and solidification. The repair process began with fixing facets, shrinking wrapping, and smoothing facets. Geometries were converted to solid, and a second repair process was applied to fix curves, edges, gaps, bad faces, missing faces, merge faces, remove small faces, and simplify complex faces and curves. In “Space Claim” program, each rib was segmented to outer cortical bone with thickness of 0.5-1 mm and inner volume from cancellous bone, based on computed tomography (CT) and Micro-CT [39].

**Fig. 2:**
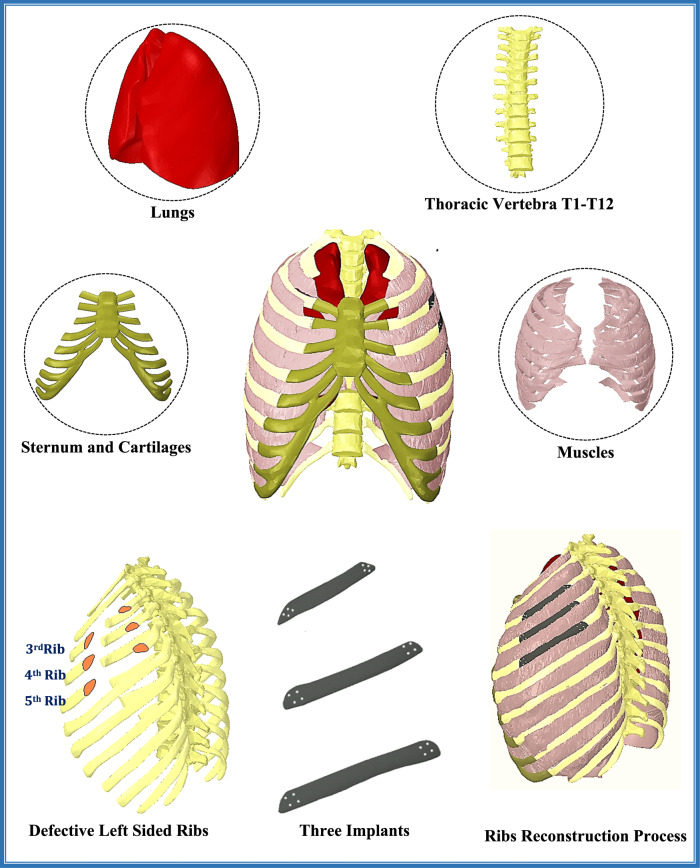
3D anatomical model of the thoracic cage and ribs reconstruction process, illustrating the lungs, thoracic vertebrae, sternum, muscles, defective ribs, and designed implants

To simulate an unhealthy case, a large defect was created in the left third, fourth, and fifth ribs, resulting in the removal of these portions. To design customized implants and replicate a realistic scenario, the chest model was made symmetrical along its mid-sagittal plane, and the missing portions of the left ribs were mirrored from the right ones. Each implant was attached to the rib with four screws on each side, as shown in Fig. 2. Therefore, a total of 24 screws with an 11 mm length and 1 mm radius were used for fixation.

After constructing all parts (rib cage, implants, muscles, and lungs), the model was assembled and exported to the ANSYS program (ANSYS 18.1, Houston, TX, USA) for computations. In ANSYS, the implants and screws were assumed to be fully bonded with ribs via their connection surfaces, with no relative sliding or motion under loading conditions. Additionally, the ribs and cartilages were in bonded connections. Figure2 displays the final model after rib cage reconstruction process.

**Fig. 3:**
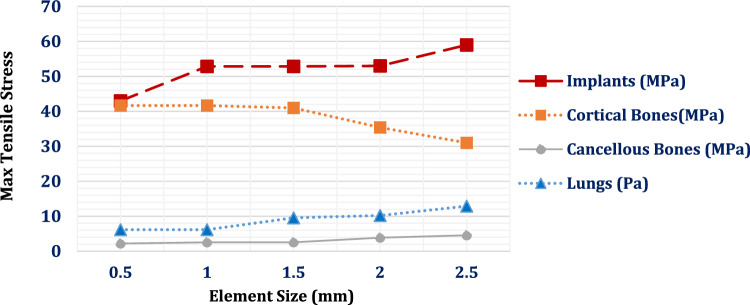
Convergence Test: The relationship between finite element mesh size (mm) and the maximum tensile stresses in titanium implants, as well as in cortical and cancellous bone of the ribs and lung tissue, was evaluated under a lateral impact load (F₁)

One of the most crucial phases in conducting a precise simulation was meshing. In ANSYS, a large mesh of elements and nodes was generated using the “Adaptive” size function with a tetrahedral mesh and element sizes ranging from 0.5 to 1 mm (Table 1). A convergence test was carried-out first to determine mesh refinement. Figure 3 illustrates the effect of finite element mesh size (0.5–2.5 mm) on the maximum tensile stresses in titanium implants, as well as in cortical bone, cancellous bone, and lung tissue, under a lateral impact load (F₁ = 1000 N) applied directly to the three implants along the negative X-axis. The same mesh setup was used for the loading condition of cardiopulmonary resuscitation manoeuvres (F_2_ *=* 600 N) to maintain consistency across finite element investigations.

**Table 1:** The number of elements and nodes used in meshing

	No. of Elements	No. of Nodes
Lungs	905,384	12,159,833
Ribs	185,004	1,122,377
Cartilages & Sternum	66,227	124,257
Muscles	1,200,239	8,234,962
Thoracic Vertebrae	93,923	170,842
Three Implants	19,949	66,775

#### Static analysis

**Fig. 4:**
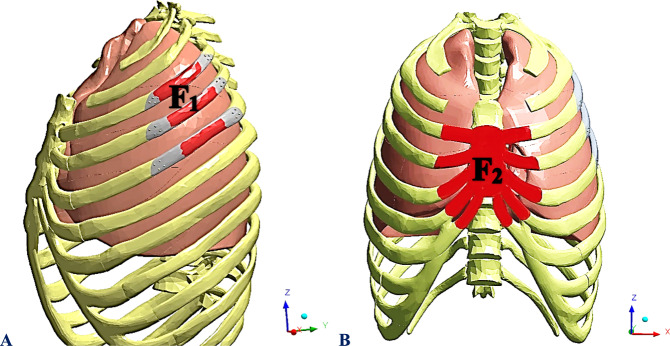
Two loading conditions: **A** Lateral force; F_1_ and **B** Sternal force; F_2_

In a typical breathing pattern, the diaphragm descends, increasing the volume of the pleural cavity, expanding the chest, and dropping chest pressure during inhalation. This process reverses during exhalation, with chest pressure increasing and pleural cavity volume decreasing [40]. Therefore, for static analysis, an extreme maximum thoracic pressure of 20 kPa was applied to the internal surfaces of the ribs and sternum. Additionally, in this study, two static forces (F_1_ and F_2_) were applied to simulate different loading conditions (Fig. 4) [41].

The impact force from a car collision can directly damage the rib cage [3, 4]. To assess the efficiency of implants in withstanding high impact, a lateral force (F_1_) of 1000 N was applied directly to the three implants in the opposite direction of the X-axis, simulating the first loading condition of a car accident.

The second condition simulated cardiopulmonary resuscitation manoeuvres by applying a sternal force (F_2_) of 600 N to the sternum and surrounding cartilages in the direction of the Y-axis [42, 43]. Cardiopulmonary resuscitation (CPR) manoeuvres are crucial in cardiac arrest (CA) to increase the chances of survival during the initial minutes of a patient’s illness [44–46]. In terms of boundary conditions, constraints were applied in the rib–vertebral connections to prevent the model from moving when the forces were applied [41, 42].

#### Mechanical properties of chest and implants

The properties of the materials considered for this research are listed in Table 2. Ribs are known for having anisotropic mechanical properties due to their intricate structure. Previous research [47, 48] have shown that with high enough image resolution (a few tens of micrometres), it is possible to evaluate a map of the bone’s anisotropic directions from CT scans. However, adult ribcage micro-CT scans can only be obtained after death due to the high radiation levels involved. Consequently, ribs have typically been studied using isotropic properties [41, 43]. Forman et al. [49] combined a hyperelastic, non-isotropic constitutive model with a linear, isotropic elastic model to represent cartilaginous tissue. Their findings indicated that the linear model, commonly used in ribcage numerical simulations, effectively represents the mechanical behaviour of cartilage. Additionally, the lung can be considered as isotropic material since its characteristics are independent of specific preferential directions, according to [50]. Therefore, linear characteristics were assumed, and material properties were applied based on earlier research to simplify the analysis and reduce calculation times [51–58].

**Table 2:** The properties of the chest parts and implants

	Elastic Modulus (GPa)	Poisson’s Ratio	Density (Kg/m3)	Tensile Strength (MPa)	Compressive Strength (MPa)	Ref
Lungs	1e-6	0.30	394	0.3e-3	0.8e-3	[52, 53]
Ribs						
Cortical Bone	13.8	0.26	2000	90–170	180–200	[6, 15, 54]
Cancellous Bone	345 e-3	0.31	1000	10	16–20	[6, 15, 54]
Cartilages	24.5 e-3	0.40	1500	5.6	8.3	[55, 56]
Muscles	17.5 e-6	0.30	1056	-	-	[57, 58]
Implants						
Titanium	90–114	0.34	3600–4500	700–862	650–848	[6, 21]
PEEK	2–3.5	0.40	1000–1300	105–140	105–140	[19, 20]
CFP60%	120–150	0.35	1300–1600	1600–2000	600–800	[23]
CFP30%	26–28	0.40	1150–1400	200–265	250–320	[59]
GFP60%	20–25	0.40	1510–1830	180–240	240–310	[60]
GFP30%	13–14	0.40	1200–1520	160–195	180–250	[61]
HAP60%	10–12.5	0.38	1900–2328	25–32	60–75	[26–28]
HAP30%	4–4.5	0.27	1700–2124	45–55	65–80	[26–28]

In this study, the implants used for reconstructing the defective ribs included traditional titanium, pure PEEK, PEEK composites reinforced with carbon or glass fibers (carbon fiber-reinforced PEEK 30% and 60%, and glass fiber-reinforced PEEK 30% and 60%), and PEEK composites containing hydroxyapatite fillers (hydroxyapatite-reinforced PEEK 30% and 60%). All fixation screws were fabricated from titanium. The material properties—including density, elastic modulus, Poisson’s ratio, and tensile and compressive yield strengths—are summarized in Table 2 [6, 19–21, 23, 26–28, 59–61]. For each implant material, the FEM results presented in this research corresponded to simulations using the maximum reported values of elastic modulus and density. A full range of material property values was compiled in Table 2 to facilitate the training of AI-based predictive models across multiple scenarios (Table 3).

**Table 3:** Validation study with the Suazo et al. study

	Compression Depth(cm)		Maximum von Mises stress (MPa)	
	Current Model	Suazo et al. [43] model	Current Model	Suazo et al. [43] model
**Z1**	5.01	5.1	32.9	35
**Z2**	6.22	6.3	41	39
**Z3**	7.14	7.5	75.78	72
**Z4**	6.59	7	57.99	61
**Z5**	7.82	8	62.54	65

#### Solving the finite element model

For evaluation, the ANSYS program extracted and analysed the maximum von Mises stresses, maximum deformations, and maximum tensile and compressive stresses and strains. The von Mises stresses were extracted to determine the relationship between implant properties and the generated stresses on ribs and lungs. Additionally, the maximum deformations were computed to assess any changes in shapes or sizes of all parts due to the applied loads.

To evaluate the durability of implants made from various materials, the maximum tensile stresses (peak maximum principal stresses) and maximum compressive stresses (peak negative minimum principal stresses) were extracted and compared to the tensile and compressive yield limits based on the principal stress theory (Rankine theory) [62, 63]. According to the Rankine theory, a complex system will fail if its maximum tensile stress surpasses the yield limit in a tension test or if its maximum compressive stress exceeds the yield limit in a compression test.

For biological components such as ribs and lungs, the maximum tensile and compressive stresses were analysed to assess their responses by comparing them to the respective yield limits (Table 2). Additionally, the tensile and compressive strains were examined and compared to acceptable limits, as excessive strains can lead to micro damage. In the case of ribs, cortical bone is damaged when the strain exceeds 2500 με in tension or 4000-5000 με in compression [15, 64]. Furthermore, cancellous bone is damaged when the strain surpasses 7000 με in tension or 8000 με in compression [15, 65]. For lungs, the permissible limits are approximately 250,000-300,000 με in tension and compression, as lungs can endure high strains before failure due to their ductility [52, 53].

### Artificial intelligence mechanical deformation prediction models

#### Data set generation

To construct a comprehensive dataset for training artificial intelligence models, finite element simulations were performed across a range of material properties (as detailed in Table 2) and loading conditions. The following forces were applied to simulate real-world scenarios:

- **Lateral Force (F**_**1**_**):** The lateral impact force, simulating trauma or accidents, varied between **500** **N and 1500** **N**, with increments of **250** **N**. This range was selected to cover mild to severe impact scenarios relevant to chest reconstruction processes.
- **Sternal Force (F**_**2**_**):** The sternal compression force, replicating conditions during cardiopulmonary resuscitation (CPR), ranged from **400** **N to 800** **N**, with increments of **100** **N**. This force range aligns with the typical chest compression forces applied during CPR maneuvers.

For each combination of forces and material properties, the finite element model computed mechanical responses such as von Mises stress, tensile and compressive stresses and strains, and total deformation across implants, ribs, and lungs. These outputs served as the dependent variables in regression modelling.

#### Machine learning and deep learning regression models

Machine learning (ML) and deep learning (DL) regression models were employed to predict the mechanical responses of rib implants (e.g., stresses, strains, and deformations) based on finite element analysis (FEA) data. These models enable accurate predictions while significantly reducing the computational burden of repeated FEA simulations. The dataset was generated through simulations under varying loading conditions and material properties, serving as the foundation for training and evaluating the predictive models. Below, the selected regression techniques are detailed, with mathematical equations and their relevance to the study.

##### Linear Regression (LR)

Linear regression, one of the simplest yet effective regression techniques, was used as a baseline model in this study. It establishes a linear relationship between the dependent variable (e.g., von Mises stress, tensile strain) and independent variables ${x}_{1},{x}_{2},\ldots ,{x}_{p}$ (e.g., material properties, loading magnitudes). The model is expressed as [66]:

1 $$y={\beta }_{0}+\mathop{\sum }\limits_{j=1}^{p}{\beta }_{j}{x}_{j}+\epsilon$$

where ${\beta }_{0}$ is the intercept, ${\beta }_{j}$ are the coefficients indicating the contribution of each predictor ${x}_{j},$ and $\epsilon$ is the residual error.

In this study, $y$ represents the mechanical responses extracted from FEA (e.g., maximum von Mises stress on ribs or implants), while the predictors ${x}_{j}$ include material properties such as elastic modulus, density, tensile strength, and loading conditions (F_1_ and F_2_). The model parameters ${\beta }_{j}$ were optimized by minimizing the Mean Squared Error (MSE) [67]:

2 $${MSE}=\frac{1}{n}\mathop{\sum }\limits_{i=1}^{n}({y}_{i}-\hat{{y}_{i}})$$

where ${y}_{i}$ is the actual response, and $\hat{{y}_{i}}$ is the predicted response for sample $i$. Linear regression provided initial insights into how material properties and loading conditions influenced mechanical responses.

##### Ridge Regression (RR)

Ridge regression was used to address multicollinearity among predictors, which can arise due to the correlated nature of material properties (e.g., elastic modulus and tensile strength). By introducing L2 -regularization, Ridge regression penalizes large coefficients to prevent overfitting. The cost function is defined as [68]:

3 $${Cost\; Function}=\frac{1}{n}\mathop{\sum }\limits_{i=1}^{n}{\left({y}_{i}-\hat{{y}_{i}}\right)}^{2}+\lambda \mathop{\sum }\limits_{j=1}^{p}{\beta }_{j}^{2}$$

where $\lambda$ is the regularization parameter that controls the penalty’s strength. A higher $\lambda$ value reduces the magnitude of ${\beta }_{j}$, shrinking coefficients for less relevant predictors.

For this study, Ridge regression was crucial when evaluating datasets with high-dimensional material properties, ensuring that predictions remained stable and interpretable, even when some properties were strongly correlated.

##### Support Vector Regression (SVR) with Radial Basis Function (RBF) Kernel

Support Vector Regression (SVR) was employed to capture non-linear relationships between material properties, loading conditions, and mechanical responses. Unlike linear models, SVR maps the input space into a higher-dimensional feature space using a kernel function. The RBF kernel, commonly used for non-linear regression, is defined as [69]:

4 $$K\left({x}_{i},{x}_{j}\right)=\exp \left(-\frac{\mathrm{||}{x}_{i}-{x}_{j}{|}{|}}{2{\sigma }^{2}}\right)$$

where $\sigma$ controls the kernel width, determining the complexity of the mapping.

SVR optimizes a cost function that minimizes prediction errors within a margin ϵ\epsilonϵ, while penalizing deviations using slack variables. This made SVR particularly effective for modeling complex mechanical behaviors (e.g., stress distributions) that could not be adequately captured by linear models.

##### Decision Tree Regression(DTR)

Decision Trees were used as an interpretable model to partition the input space based on material properties and loading conditions. The algorithm recursively splits the data into subsets, minimizing the variance in the target variable at each node. The variance reduction for a split is given by [70]:

5 $${Variance\; Reduction}={Var}\left(y\right)-\left(\frac{nL}{n}Var\left(yL\right)+\frac{nR}{n}Var(yR)\right)$$

where ${Var}\left(y\right)$ is the variance of the target variable, and *nL*, *nR*, and *n* are the sizes of the left, right, and parent nodes, respectively.

For this study, Decision Trees provided insights into the hierarchical importance of input features (e.g., loading conditions vs. material properties) on mechanical responses. These insights guided the interpretation of more complex ensemble models.

##### Random Forest Regression (RFR)

Random Forests extend Decision Trees by combining predictions from multiple trees to improve accuracy and robustness. Each tree is trained on a bootstrapped subset of the dataset, and predictions are aggregated as [71, 72]:

6 $$\hat{y}=\frac{1}{{N}_{t}}\mathop{\sum }\limits_{t=1}^{{N}_{t}}\hat{{y}_{t}}$$

where ${N}_{t}$ is the number of trees, and $\hat{{y}_{t}}$ is the prediction from the $t$-th tree.

In this study, Random Forests were particularly effective in capturing non-linear interactions between material properties and loading conditions. Additionally, the ensemble approach mitigated overfitting and improved generalization, especially for outliers in the FEA dataset.

##### Fully Connected Neural Networks (FCN)

Fully Connected Neural Networks (FCNs) were used to model complex, non-linear relationships between input features and mechanical responses. Each neuron in the network computes [73, 74]:

7 $${z}^{(l)}={W}^{(l)}{a}^{(l-1)}+{b}^{(l)}$$

8 $${a}^{\left(l\right)}=f({z}^{\left(l\right)})$$

where ${W}^{(l)}$ and ${b}^{(l)}$ are the weights and biases for layer $l$ and $f$ is an activation function (e.g., ReLU, sigmoid).

For this study, FCNs were trained using backpropagation to minimize the MSE. They excelled at learning complex patterns in the high-dimensional dataset, such as stress concentrations in specific implant regions.

##### Deep Fully Connected Neural Networks (DFCN)

Deep FCNs extend basic FCNs by incorporating additional hidden layers to enhance the network’s capacity to learn hierarchical feature representations. Regularization techniques, such as dropout, were employed to prevent overfitting, ensuring that predictions generalized well across unseen data [75].

##### LightGBM Regression (LGBM R)

LightGBM, a gradient boosting framework, was employed for its efficiency and scalability. The model optimizes an objective function [76]:

9 $${Objective}=\mathop{\sum }\limits_{i=1}^{n}{\mathcal{l}}\left({y}_{i},\hat{{y}_{i}}\right)+\varOmega (f)$$

where ${\mathcal{l}}\left({y}_{i},\hat{{y}_{i}}\right)$ is the loss function (e.g., MSE), and $\varOmega \left(f\right)$ is a regularization term. By using histogram-based techniques for data splitting, LightGBM was able to oversee the large and diverse FEA dataset with minimal computational overhead.

#### Prediction model evaluation

Model evaluation is a critical step in assessing the performance of predictive models, particularly for regression tasks and the prediction of mechanical responses. Several key metrics are commonly used to quantify both the accuracy and reliability of these models. The following provides a detailed explanation of each metric, along with their corresponding mathematical formulations:

##### Coefficient of Determination (R²)

The Coefficient of Determination, or R^2^, is a statistical measure that represents the proportion of the variance in the dependent variable that is predictable from the independent variables. It ranges from 0 to 1, with 1 indicating perfect prediction. It is calculated as [67]:

10 $${R}^{2}=1-\frac{{\sum }_{i=1}^{n}{\left({y}_{i}-\hat{{y}_{i}}\right)}^{2}}{{\sum }_{i=1}^{n}{\left({y}_{i}-\hat{{y}_{i}}\right)}^{2}}$$

where ${y}_{i}$ represents the actual values, $\hat{{y}_{i}}$ represents the predicted values, and yˉ\bar{y}yˉ is the mean of the actual values. A higher R² indicates a better model fit, with values closer to 1 indicating that the model explains more of the variance in the data.

##### Mean Absolute Error (MAE)

The Mean Absolute Error quantifies the average magnitude of the errors in a set of predictions, without considering their direction (i.e., whether the errors are positive or negative). It is the average of the absolute differences between predicted and actual values, and it is computed as [67]:

11 $${MAE}=\frac{1}{n}\mathop{\sum }\limits_{i=1}^{n}\left|{y}_{i}-\hat{{y}_{i}}\right|$$

A lower MAE indicates better model accuracy, while this metric is less sensitive to outliers compared to others.

##### Mean Squared Error (MSE)

The Mean Squared Error calculates the average of the squared differences between actual and predicted values. MSE is more sensitive to large errors compared to MAE, as it squares the errors, giving more weight to larger differences and is given by Eq. 2.

##### Root Mean Squared Error (RMSE)

The Root Mean Squared Error is the square root of MSE, making it interpretable in the same units as the target variable. Like MSE, RMSE also penalizes large errors but is easier to interpret because it is in the same units as the target.

12 $${RMSE}=\sqrt{\frac{1}{n}\mathop{\sum }\limits_{i=1}^{n}{\left({y}_{i}-\hat{{y}_{i}}\right)}^{2}}$$

This metric provides the error magnitude in the same units as the target variable, making it more interpretable.

##### Execution Time

Execution time measures the time it takes for a model to make predictions or fit to the data. While this metric does not directly assess the accuracy of the model, it is a crucial factor in the practical deployment of the model, particularly for large datasets or real-time applications.

## Results

The results were divided into two parts: the results of the finite element model and the results of artificial intelligence models

### Finite element analysis

#### Validation study

Firstly, in this research, the model of an intact (healthy) chest was validated with Suazo et al. [43] model. Both models were constructed based on the CT images available in the BodyParts3D anatomy database [37, 38]. For validation, cardiopulmonary resuscitation manoeuvres were simulated using five compression zones distributed on the breastbone. Each zone was defined as a 10-cm^2^ surface patch. The first and second compression zones (Z1 and Z2) were cantered on the midline of the breastbone, while the other three compression zones (Z3, Z4, and Z5) were slightly cantered on the cartilaginous tissue of the fourth, fifth, and sixth left ribs, respectively. The rib cage’s maximum von Mises stresses (MPa) and maximum compression depth (cm) were retrieved when a force of 600 N was applied to the compression zones (Fig. 4).

By utilizing the five compression zones Z1, Z2, Z3, Z4, and Z5, the compression depth of the current model decreased by 1.76%, 1.26%, 4.8%, 5.85%, and 2.25%, respectively, compared to the findings of Suazo et al. [43]. Moreover, the maximum von Mises stress extracted was only changed by a maximum of 6% across the five compression zones.

#### Implants stresses

**Fig. 5:**
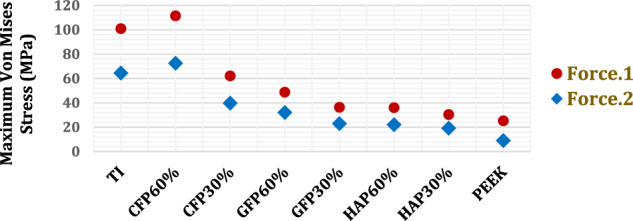
Maximum von Mises stresses (MPa) on PEEK and PEEK composites implants

**Fig. 6:**
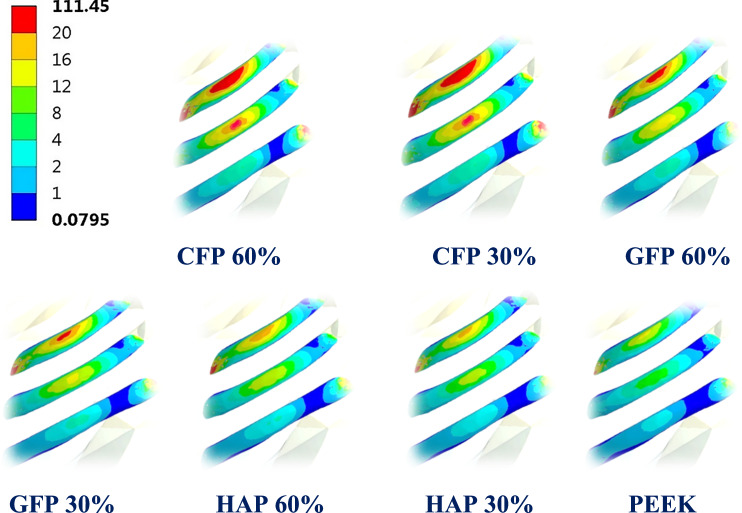
Distribution of von Mises stresses (MPa) on the PEEK and PEEK composites implants under force.1

As shown in Fig. 5 and Table 4, the maximum von Mises stresses were extracted for all implants, along with the maximum tensile and compressive stresses, to be compared with their yield strengths to evaluate each implant’s condition (endurance or failure) under the two loading conditions. Figure 6 illustrates the distribution of von Mises stresses on the PEEK composite implants under force (F_1_). As shown in Fig. 6, the upper implant was exposed to higher stresses compared to the other implants.

**Table 4:** Maximum tensile and compressive stresses (MPa) on PEEK and PEEK composites implants

	Ti	CFP 60%	CFP 30%	GFP 60%	GFP 30%	HAP 60%	HAP 30%	PEEK
Force.1								
Tensile Stress	52.86	59.03	40.22	37.83	36.15	35.27	33.47	23.21
Compressive Stress	107.67	118.21	70.66	52.31	33.93	31.78	26.05	20
Force.2								
Tensile Stress	67	75.43	41.19	33.38	23.86	22.86	19.86	9.326
Compressive Stress	31.51	35.946	16.973	13.62	9.053	8.66	8.626	6.78

Under Force 1, the use of CFP 60% implants increased the maximum von Mises stress on the implants by 10.445% compared to that on the titanium implants. However, the maximum von Mises stresses reduced by 38.45%, 51.71%, 65%, 65%, 69.9%, and 75% on CFP 30%, GFP 60%, GFP 30%, HAP 60%, HAP 30%, and PEEK, respectively. Consequently, the maximum tensile and compressive stresses increased by 11.67% and 9.78% on the CFP 60% implants and reduced by 23.9% and 34.37%, 28.43% and 51.41%, 31.61% and 68.48%, 33.27% and 70.48%, 36.68% and 75.8%, and 56.09% and 81.42% on the CFP 30%, GFP 60%, GFP 30%, HAP 60%, HAP 30%, and PEEK implants, respectively.

Under force 2, for the CFP 60% implants, the maximum von Mises stress was 12.49% greater than that on the titanium implants. Conversely, the maximum von Mises stresses reduced on CFP 30%, GFP 60%, GFP 30%, HAP 60%, HAP 30%, and PEEK by 38.34%, 50.26%, 64.39%, 65.79%, 70.28%, and 86.03%, respectively. The maximum tensile and compressive stresses increased by 12.59% and 14.07%, respectively, on the CFP 60% implants and decreased by 38.52% and 46.14%, 50.18% and 56.78%, 64.39% and 71.27%, 65.87% and 72.50%, 70.36% and 72.63%, and 86.08% and 78.49%, respectively, on the CFP 30%, GFP 60%, GFP 30%, HAP 60%, HAP 30%, and PEEK implants.

**Fig. 7:**
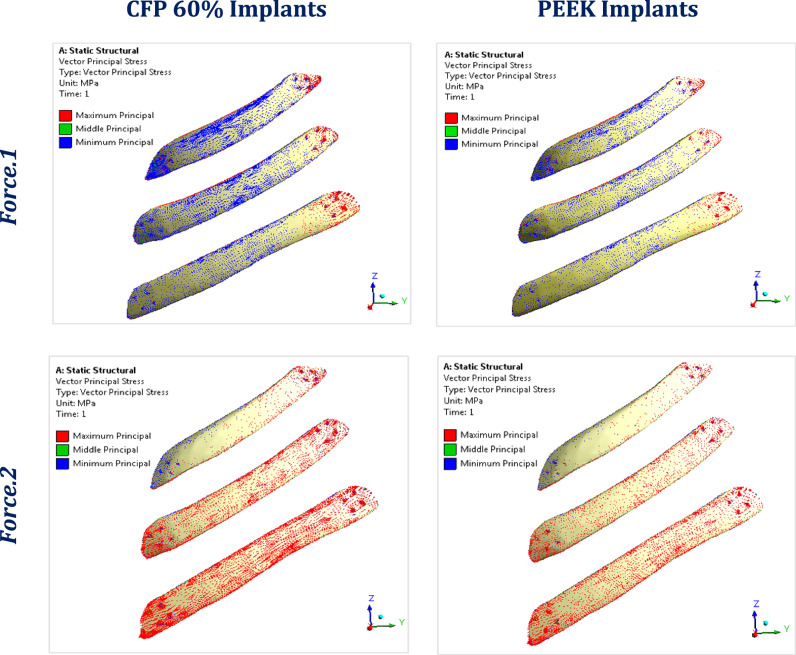
The distribution of tensile (red arrows) and compressive (blue arrows) stresses on CFP 60% and PEEK implants, under lateral impact force (F_1_) and sternal force (F_2_)

Figure 7 illustrates the principal stress vectors of the three CFP 60% and PEEK implants, under force.1 and force.2 respectively. The red and blue arrows indicated regions of tension and compression, respectively. Under lateral impact force (F_1_), compressive stresses were concentrated along the length of the implants, while tensile stresses were more concentrated in the peripheral regions around the fixation points. Additionally, higher tensile and compressive stresses were observed in CFP 60% implants compared to PEEK implants due to their higher stiffness. Under sternal force (F_2_), tensile stresses were clearly concentrated along the implants.

#### Ribs stresses and strains

**Fig. 8:**
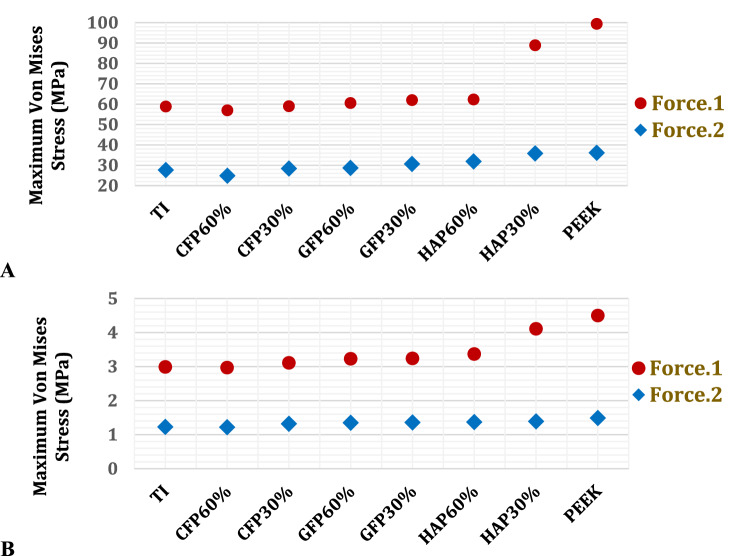
Maximum von Mises stresses (MPa) on: **A** cortical bones and **B** cancellous bones of left ribs by using PEEK and PEEK composites implants

**Fig. 9:**
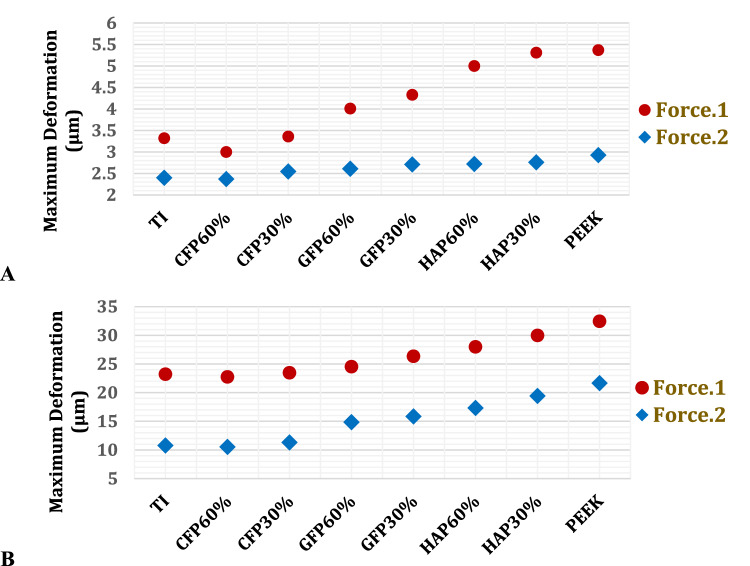
Maximum deformations (μm) on: **A** cortical bones and **B** cancellous bones of left ribs, using PEEK and PEEK composites implants

As depicted in Figs. 8 and 9, the maximum von Mises stresses were determined for the cortical and cancellous bones of the left ribs to investigate the relationship between implant properties and the stresses experienced by the ribs. Additionally, maximum deformations were analysed to assess changes in the size and shape of the ribs. Figures 8 and 9 illustrate that CFP 60% implants resulted in the lowest stresses and deformations on the cortical and cancellous bones, while PEEK implants led to the highest stresses and deformations. Furthermore, the use of CFP 30% implants caused a slight change in the stresses and deformations on the bones. In contrast, the stresses and deformations on the bones were significantly increased when using GFP 60%, GFP 30%, HAP 60%, and HAP 30% implants, compared to titanium implants.

For the cortical and cancellous bones, the maximum tensile and compressive stresses and strains were analysed to compare them with the respective yield limits and evaluate their responses, according to failure theory [62, 63]. For cortical bones, Table 5 illustrates that CFP 60% implants reduced stresses and strains on ribs compared to titanium implants under force.1 and force.2. However, CFP 30%, GFP 60%, GFP 30%, HAP 60%, HAP 30%, and PEEK implants increased stresses and strains on the ribs. Under force.1, the maximum tensile and compressive stresses increased by 10.3% and 1.69%, 14.38% and 5.5%, 20.07% and 6.52%, 20.72% and 6.89%, 22.68% and 71.2%, and 69.79% and 96.27%, respectively on cortical bones. Consequently, the maximum tensile and compressive strains increased by 10.29% and 0.27%, 14.36% and 4.03%, 20.05% and 5.03%, 20.7% and 5.41%, 22.68% and 70.21%, and 30.24% and 90.62% when using CFP 30%, GFP 60%, GFP 30%, HAP 60%, HAP 30%, and PEEK, respectively.

**Table 5:** Maximum tensile and compressive stresses and strains on the cortical bones of left ribs, using PEEK and PEEK composites implants

	Ti	CFP 60%	CFP 30%	GFP 60%	GFP 30%	HAP 60%	HAP 30%	PEEK
Force.1								
Tensile Stress (MPa)	41.65	40.68	45.94	47.64	50.01	50.28	51.1	70.72
Compressive Stress (MPa)	59	58.5	60	62.25	62.85	63.07	101.01	115.8
Tensile Strain (μԐ)	1984	1937	2188	2269	2381	2394	2434	2584
Compressive Strain(μԐ)	3324	3250	3333	3458	3492	3504	5658	6337
Force.2								
Tensile Stress (MPa)	33.26	30.21	36.94	38.38	40.32	40.54	41.14	42.62
Compressive Stress (MPa)	31.93	29.11	34.57	34.74	36.58	36.80	37.46	40.00
Tensile Strain(μԐ)	1584	1439	1759	1827	1920	1930	1959	2011
Compressive Strain(μԐ)	2128	1940	2304	2316	2438	2453	2493	2589

Table 5 illustrates that under force 2, CFP 60% implants also reduced the stresses and strains on the cortical bones of the left ribs, unlike the other implants. The decreases in the maximum tensile and compressive stresses, and the maximum tensile and compressive strains were 9.17% and 8.8%, and 9.15% and 8.8%, respectively. Conversely, the CFP 30%, GFP 60%, GFP 30%, HAP 60%, HAP 30%, and PEEK implants increased the stresses and strains on the ribs. The increases in the maximum tensile and compressive stresses were 11.06% and 8.26%, 15.39% and 8.8%, 21.2% and 14.56%, 21.8% and 15.25%, 23.69% and 17.31%, and 28.14% and 25.27%, respectively. Consequently, the maximum tensile and compressive strains increased by 11.05% and 8.27%, 15.38% and 8.83%, 21.24% and 14.56%, 21.87% and 15.27%, 23.68% and 17.15%, and 27.01% and 21.66%, respectively, when using CFP 30%, GFP 60%, GFP 30%, HAP 60%, HAP 30%, and PEEK implants.

For cancellous bones, Table 6 illustrates that CFP 60% and CFP 30% implants slightly changed the stresses and strains on the ribs under two forces (F1 and F2) compared to titanium implants. However, GFP 60%, GFP 30%, HAP 60%, HAP 30%, and PEEK implants significantly increased the stresses and strains on the ribs. Under force.1, the increases in the maximum tensile and compressive stresses were 8.63% and 3.6%, 9.41% and 4.5%, 10.59% and 5.41%, 11.37% and 6.61%, and 13.33% and 7.81%, respectively. Consequently, the maximum tensile and compressive strains increased by 1.11% and 1.46%, 1.75% and 1.7%, 2.31% and 3.63%, 2.5% and 3.83%, and 3.55% and 4.52% when using GFP60%, GFP30%, HAP60%, HAP30%, and PEEK, respectively. Under force.2, CFP 60%, CFP 30%, and GFP60% implants did not significantly change the stresses and strains on cancellous bones compared to titanium implants. However, using GFP 30%, HAP 60%, HAP 30%, and PEEK implants, the maximum tensile and compressive stresses increased by 2.56% and 1.56%, 3.39% and 2.08%, 5.05% and 3.13%, and 6.7% and 3.65%, respectively. Additionally, the maximum tensile and compressive strains increased by 0.73% and 1.60%, 1.2% and 1.73%, 1.33% and 1.78%, and 1.84% and 1.97%, respectively.

**Table 6:** Maximum tensile and compressive stresses and strains on the cancellous bones of left ribs, using PEEK and PEEK composites implants

	Ti	CFP 60%	CFP 30%	GFP 60%	GFP 30%	HAP 60%	HAP 30%	PEEK
Force.1								
Tensile Stress (MPa)	2.55	2.53	2.58	2.77	2.79	2.82	2.84	2.89
Compressive Stress (MPa)	3.33	3.23	3.43	3.45	3.48	3.51	3.55	3.59
Tensile Strain (μԐ)	6655	6650	6663	6729	6772	6809	6822	6899
Compressive Strain(μԐ)	7512	7500	7519	7622	7640	7785	7800	7852
Force.2								
Tensile Stress (MPa)	1.209	1.20	1.21	1.22	1.24	1.25	1.27	1.29
Compressive Stress (MPa)	1.92	1.91	1.922	1.93	1.95	1.96	1.98	1.99
Tensile Strain(μԐ)	3160	3157	3166	3172	3183	3198	3202	3218
Compressive Strain(μԐ)	5327	5320	5402	5409	5412	5419	5422	5432

**Fig. 10:**
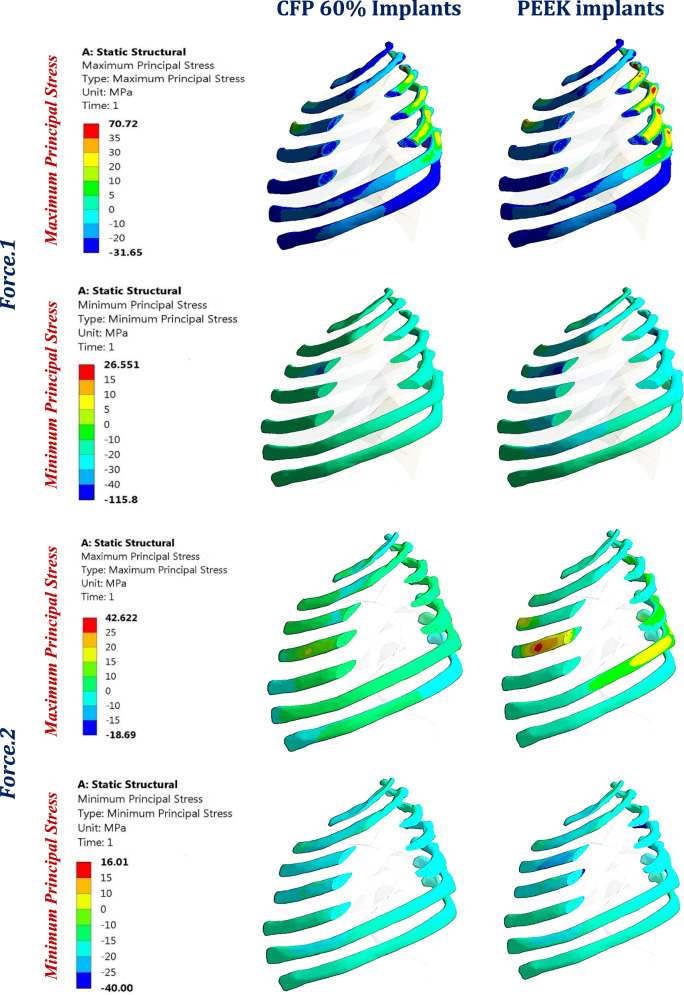
Distribution of maximum and minimum principal stresses (MPa) on the left ribs under force.1 and force.2, using CFP 60% and PEEK implants

**Fig. 11:**
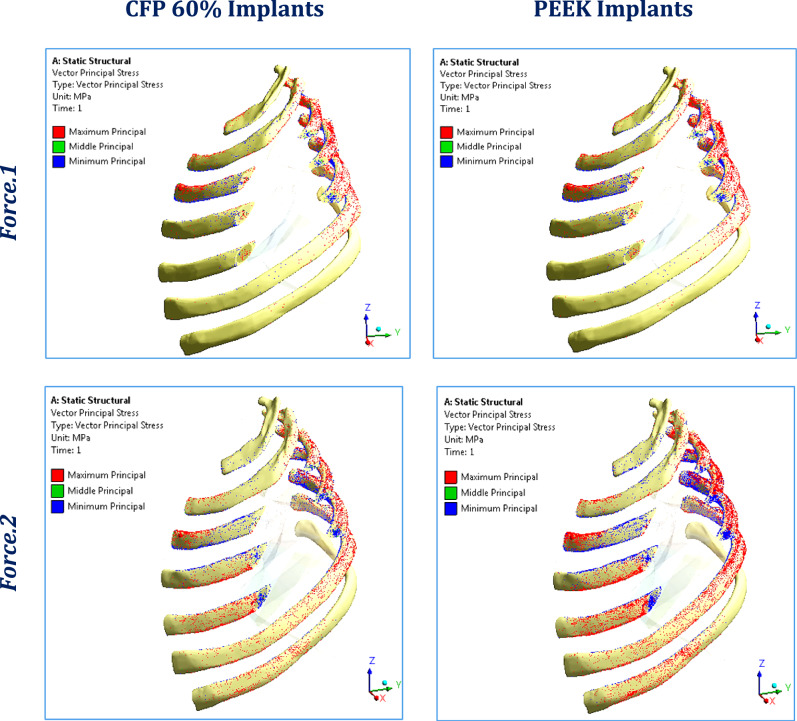
The distribution of tensile (red arrows) and compressive (blue arrows) stresses on the left ribs under force.1 and force.2, using: CFP 60% and PEEK implants

Figure 10 illustrates the distribution of maximum and minimum principal stresses on the left ribs under force.1 and force.2, using CFP 60% and PEEK implants. Figure 11 illustrates the principal stress vectors of the left ribs using CFP 60% and PEEK implants under the two forces. The figures show that tensile stresses were more concentrated on the external surfaces, while compressive stresses were more concentrated on the internals. Additionally, higher tensile and compressive stresses appeared on the left third, fourth, and fifth ribs when using PEEK implants compared to CFP 60% implants.

#### Lung stresses and strains

**Fig. 12:**
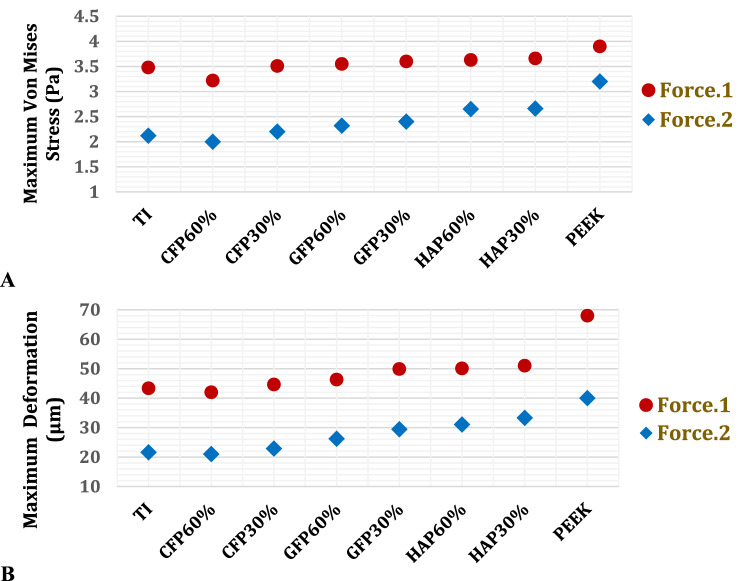
**A** Maximum von Mises stresses (Pa) and **B** Maximum deformation (μm) of the left lung, using PEEK and PEEK composites implants

**Fig. 13:**
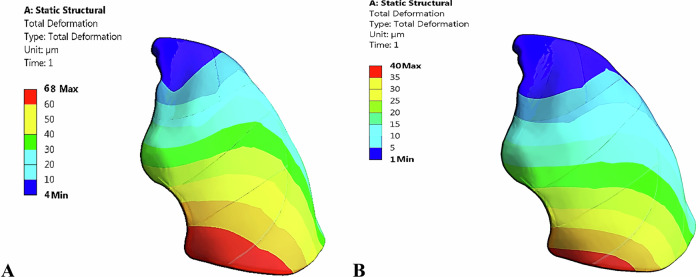
Distribution of total deformation on left lung by using PEEK implants, under: **A** force.1 and **B** force.2

This section aimed to determine the maximum tensile and compressive stresses and strains of the left lung, as well as the von Mises stresses and deformations. As shown in Fig.12 and Table 7, the CFP 60% implants reduced stresses, strains, and deformations on the lung compared to titanium implants, unlike other polymeric implants. Figure 13 illustrates the distribution of total deformation on the left lung using PEEK implants under force.1 and force.2, respectively. The maximum values appeared in the lower-right regions, while the minimum values appeared in the upper-left regions. As shown in the Fig. 12, lung deformation increased by 2.9%, 6.8%, 15.07%, 15.557%, 17.67%, and 56.812% with the CFP 30%, GFP 60%, GFP 30%, HAP 60%, HAP 30%, and PEEK implants, respectively, under force.1. However, lung deformation reduced by 3.14% with the CFP 60% implants. Under force.2, lung deformation reduced by 2.77% with the use of CFP 60%, but increased by 5.857%, 21.208%, 36.190%, 43.650%, 54%, and 84.9% with the use of CFP 30%, GFP 60%, GFP 30%, HAP 60%, HAP 30%, and PEEK implants, respectively.

**Table 7:** Maximum tensile and compressive stresses and strains on the left lung, using PEEK and PEEK composites implants

	Ti	CFP 60%	CFP 30%	GFP 60%	GFP 30%	HAP 60%	HAP 30%	PEEK
Force.1								
Tensile Stress (Pa)	6.16	6.11	6.21	6.34	6.46	6.49	6.52	7.4
Compressive Stress (Pa)	3.17	3.02	3.477	3.85	4.2	4.29	6.199	7.4
Tensile Strain (μԐ)	6164	6139	6210	6344	6467	6492	6523	7489
Compressive Strain(μԐ)	3177	3022	3479	3850	4200	4297	6199	7456
Force.2								
Tensile Stress (Pa)	3.88	3.43	3.9	4.2	4.38	4.39	5.2	5.8
Compressive Stress (Pa)	2.7	2.3	2.9	3.2	3.92	4	5.3	5.82
Tensile Strain(μԐ)	3833	3434	3978	4200	4320	4323	5250	5800
Compressive Strain(μԐ)	2720	2321	2987	3245	3978	4000	5349	5845

Under force 1, the use of CFP 60% implants resulted in a nearly 7.47% reduction in the maximum von Mises stress on the lung. As a result, the maximum tensile and compressive stresses decreased by 0.812% and 4.73%, respectively, and the maximum tensile and compressive strains decreased by 0.4% and 4.87%, respectively. The maximum von Mises stress and the maximum tensile stress and strain of the CFR 30% implants were almost unchanged compared to those of the titanium implants. However, the maximum compressive stress and strain increased by nearly 9.6%. An increase in the maximum von Mises stress, as well as the maximum tensile and compressive stresses and strains, was observed for the GFP 60% and CFP 30% implants. With the HAP 60% and HAP 30% implants, the maximum von Mises stresses increased by 4.3% and 5.17%, respectively. The maximum tensile and compressive stresses increased by 5.35% and 35.331%, and 5.8% and 95.55%, respectively, while the maximum tensile and compressive strains increased by 5.32% and 35.25%, and 5.8% and 95%, respectively. Compared to other materials, PEEK implants produced the highest stresses and strains on the lungs. The maximum von Mises stress, as well as the maximum tensile stress and strain, increased by 12.06%, 20.13%, and 21.49%, respectively, with the use of PEEK implants. Additionally, the maximum compressive stress and strain were nearly doubled compared to those of titanium implants.

Under force 2, with the CFP 60% implants, the maximum von Mises stress on the lung reduced by 5.6% compared to that with the titanium implants. Hence, the maximum tensile and compressive stresses and strains reduced by 11.598% and 14.815%, and by 10.410% and 14.66%, respectively. The CFP 30% implants increased the maximum von Mises stress on the lung by 3.774% and the maximum tensile and compressive stresses and strains by 0.515% and 7.4%, and 3.78% and 9.81%, respectively. The maximum von Mises stress increased by 9.434% and 13.2% in the GFP 60% and GFP 30% implants, respectively. Consequently, the maximum tensile and compressive stresses increased by 8.247% and 18.519%, and 12.887% and 45.185%, and the maximum tensile and compressive strains increased by 9.575% and 19.301%, and 12.705% and 46.250%, respectively. An increase in the maximum von Mises stress was also observed using the HAP 60% and HAP 30% implants, with increases of 25% and 25.47%, respectively. Consequently, the maximum tensile and compressive stresses increased by 13.14% and 48.14%, and by 59.79% and 118.5%, respectively. Additionally, the maximum tensile and compressive strains increased by 12.784% and 47.05%, and by 63.05% and 96.65%, respectively. The use of PEEK implants resulted in significant increases in the maximum von Mises stress, maximum tensile stress, maximum compressive stress, maximum tensile strain, and maximum compressive strain by 50.94%, 49.48%, 133.3%, 51.3%, and 133.27%, respectively.

**Fig. 14:**
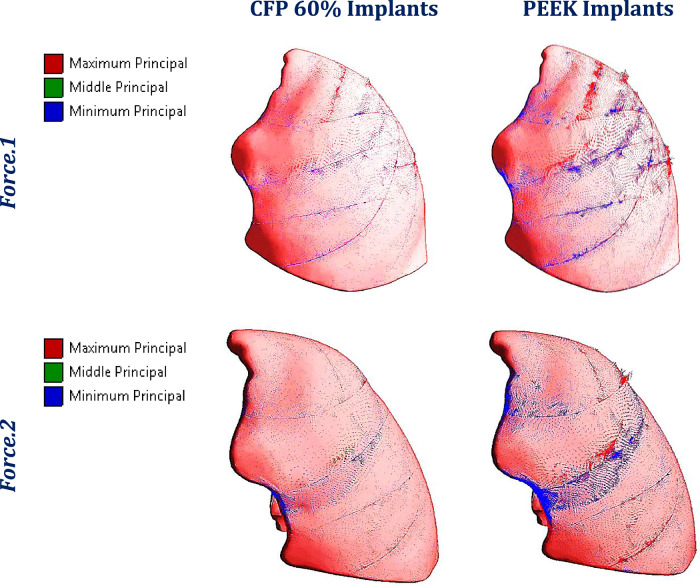
Principal stress vectors for the left lung under force.1 and force.2, using CFP 60% and PEEK implants

Figure 14 depicts the principal stress vectors for the left lung under force.1 and force.2 using CFP 60% and PEEK implants. The red and blue arrows represent areas of tension and compression, respectively. In both force.1 and force.2 conditions, the majority of stresses were compressive near the loaded areas. Furthermore, tensile and compressive stresses around these areas were more focused using the PEEK implants compared to the CFP 60% implants, where stresses are evenly spread out.

### Artificial intelligence

The integration of AI with FEA aimed to create predictive models capable of estimating mechanical responses—stresses, strains, and deformations—for rib implants under varied loading conditions. A comprehensive dataset was constructed using FEA simulations, incorporating material properties, force directions, magnitudes, and corresponding mechanical outcomes.

Based on the outlined results in Table 8 and Table 9, Linear and Ridge Regression models emerged as top performers in predicting mechanical responses. These models achieved perfect or near-perfect R² values, with linear regression consistently achieving R² = 1.0 under Force 1 and near-perfect accuracy (R² = 0.9999) under Force 2. Minimal error rates were observed, with MAE and RMSE as low as 1.85 and 4.45, respectively, under Force 1. The exceptional computational efficiency of these models was evident as predictions were completed in microseconds (∼0.0002 s), making them ideal for real-time applications. Table 8 consolidates performance metrics under Force 1.

**Table 8:** Evaluation metrics of AI regression models under Force 1

Regression Model	R2	MAE	MSE	RMSE	Accuracy	Execution Time
Linear Regression	1.000	1.8543	19.8279	4.4529	0.9998	0.0002
Ridge Regression	1.000	1.8696	19.9144	4.4626	0.9998	0.0002
SVR_RBF	0.999	7.8982	511.1149	22.6078	0.9990	1.2764
Decision Tree	0.999	9.2780	489.3882	22.1221	0.9988	0.0429
Random Forest	0.999	9.2199	489.7667	22.1307	0.9988	1.2525
Fully Connected Network	1.000	4.9880	141.0645	11.8771	0.9994	1.6617
Deep Fully Connected Network	1.000	3.1165	191.0513	13.8221	0.9996	3.7105
LightGBM Regression	1.0000	3.1165	191.0513	13.8221	0.9996	0.0002

**Table 9:** Evaluation metrics of AI regression models under Force2

Regression Model	R2	MAE	MSE	RMSE	Accuracy	Execution Time
Linear Regression	0.9999	3.7503	249.5266	15.7964	0.9994	0.0003
Ridge Regression	0.9999	3.7947	249.7796	15.8044	0.9994	0.0003
SVR_RBF	0.9993	12.8835	3174.5000	56.3431	0.9979	0.0932
Decision Tree	0.9994	17.1422	2634.6000	51.3287	0.9972	0.0541
Random Forest	0.9994	17.5605	2646.3000	51.4417	0.9971	1.1057
Fully Connected Network	0.9999	3.9052	589.0385	24.2701	0.9994	1.1714
Deep Fully Connected Network	1.0000	2.3264	62.8125	7.9254	0.9996	1.2894
LightGBM Regression	1.0000	2.3264	62.8125	7.9254	0.9996	0.0002

The SVR model showcased high accuracy (R² ≥ 0.9993), particularly in capturing non-linear relationships within the dataset. However, the limitations included higher error metrics, with MAE ranging from 7.89 to 12.88 and RMSE reaching up to 56.34 under Force 2, as detailed in Table 8. Additionally, SVR exhibited the longest computational times (*≥*1.27 s under Force 1), making it less ideal for applications requiring rapid processing.

Decision Tree Regression offered interpretable predictions, achieving competitive R² values consistently above 0.9994 (Tables 8 and 9). Error rates were moderate, with MAE and RMSE values around 9.28 and 22.12 under Force 1. Predictions were completed in approximately 0.05 seconds, though still slower than linear models. Visual comparisons of the feature importance of rankings emphasize the hierarchical role of material properties and loading conditions in influencing mechanical responses.

Neural network models demonstrated their strength in handling complex, high-dimensional datasets. Both FCN and Deep FCN models achieved R² values close to or equal to 1.0, indicating exceptional performance in capturing non-linear patterns (Tables 8 and 9). MAE values ranged from 2.32 to 4.99, with RMSE as low as 7.92 for Deep FCN under Force 2. However, predictions required more time (1.17–3.71 s; Table 8).

The LightGBM model emerged as the most efficient advanced regression technique, achieving flawless accuracy (R² = 1.0 across both force conditions, as shown in Tables 7 and 2). LightGBM achieved low error rates (MAE as low as 2.32 and RMSE as low as 7.92 under Force 2) and demonstrated exceptional efficiency, completing predictions in less than 0.0002 seconds.

Linear Regression and Ridge Regression proved to be the most effective models for datasets with linear dependencies, offering a balance of high accuracy, minimal error rates, and unparalleled computational speed. For datasets with non-linear relationships, LightGBM Regression stood out as the optimal choice due to its ability to combine the predictive power of deep learning models with computational efficiency. Although SVR captured non-linear relationships effectively, its computational demands and higher error rates limit its practicality. Decision Trees, while interpretable, exhibited higher error rates compared to other models, suggesting their suitability for exploratory analysis rather than precise predictions. Advanced deep learning models, such as FCN and Deep FCN, excelled in capturing intricate patterns but are better suited for scenarios prioritizing prediction complexity over execution time constraints.

**Fig. 15:**
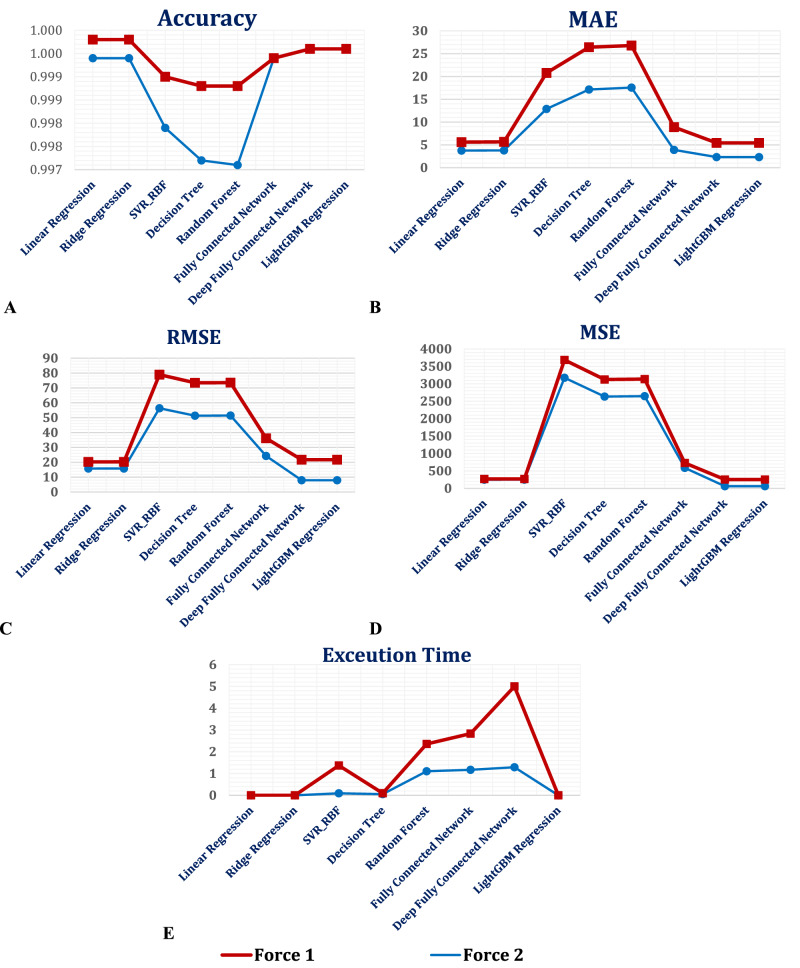
Comparisons Across Models and Force Conditions: **A** Accuracy (R²), **B** Mean Absolute Error (MAE), **C** Root Mean Squared Error (RMSE), **D** Mean Squared Error (MSE), and **E** Execution Time

The comparative analysis of regression models under the two different forces, as visualized in Fig. 15, revealed significant insights into model performance. Each figure reflected key aspects of model evaluation, such as predictive accuracy, error rates, and computational efficiency, offering a comprehensive understanding of their strengths and limitations.

Figure 15A presents the accuracy (R^2^) across models, demonstrating the models’ ability to explain the variability in the target data. Linear Regression, Ridge Regression, and LightGBM exhibit near-perfect R² values, with scores consistently close to 1.0 across both force conditions. This underscores their robustness in handling both linear and complex relationships within the dataset. Conversely, models such as SVR and Decision Trees exhibited slightly lower accuracy under Force 2, indicating their reduced ability to generalize in certain scenarios.

Figure 15B highlights the MAE as a measure of average prediction error magnitude. Linear Regression, Ridge Regression, and LightGBM demonstrated minimal MAE values across both conditions, confirming their superior predictive accuracy. Neural networks (Fully Connected Network and Deep Fully Connected Network) also achieved commendable performance but with marginally higher errors compared to LightGBM. In contrast, models like SVR and Decision Trees exhibited significantly elevated MAE values, particularly under Force 2, indicating a higher degree of deviation from actual values.

The RMSE, shown in Fig. 15C, provided further evidence of prediction precision. Like MAE trends, LightGBM, Linear Regression, and Ridge Regression achieved the lowest RMSE values, reinforcing their reliability in diverse scenarios. On the other hand, SVR and Random Forest models displayed noticeably higher RMSE values, suggesting weaker predictive capabilities in scenarios involving Force 2. As depicted in Fig. 15D, the MSE underscores the consistency of model performance. LightGBM emerged as the standout model with minimal MSE values across both conditions, reflecting low prediction variability. Models such as SVR and Decision Trees, however, showed substantially higher MSE values, especially under Force 1, indicating their limited capacity to minimize error variance.

Figure 15E emphasizes computational efficiency, which is critical for real-time applications. Linear Regression, Ridge Regression, and LightGBM were the fastest models, completing predictions in under 0.0002 seconds. This made them ideal for applications requiring immediate responses. In contrast, neural networks (Fully Connected Network and Deep Fully Connected Network) and SVR exhibited considerably longer execution times, exceeding one second in some cases. Although these models exceled in accuracy, their computational demands limited their suitability for time-sensitive tasks.

## Discussion

Resections of the rib cage due to malignancies, locally invasive cancers, metastatic lesions, car crashes, or infections are the most common causes of chest defects [1, 2]. Skeletal restoration using biological, alloplastic, or synthetic materials is usually necessary for defects larger than 5 cm in the anterior chest, over 10 cm in the posterior chest, or involving more than 3 ribs, regardless of location [77]. Restoring the skeletal stability and stiffness of the chest wall is known as rib cage reconstruction process. This process is essential for protecting the intrathoracic organs and preserving breathing mechanics [3]. Ribs are more susceptible to damage than any other thoracic bone, making surgical rib fixation and reconstruction using plates and implants common treatments [4]. However, this repair remains debatable regarding the procedure and the type of material or implant to be used [78]. Furthermore, the primary criteria to consider are the defect’s position and size that require reconstruction [79].

A biomimetic prosthetic rib, resembling a natural rib, must be designed and manufactured to repair defects in the rib cage [1–3]. The advancement of Additive Manufacturing (AM) technology has enabled the creation of customized implants for rib cage reconstructions. By utilizing the patient’s computed tomography (CT) data, an implant model that accurately reflects the patient’s anatomy can be constructed based on the precise location and dimensions of the chest resection [80, 81].

A variety of materials, including pig dermis, AlloDerm, and bovine pericardium, as well as synthetic alloplastic and metallic materials, have been used in rib reconstruction [2]. Currently, artificial ribs are frequently made of metallic materials such as titanium alloys, cobalt-based alloys, pure titanium, and stainless steel [5–7]. Metals have high biocompatibility, durability, strength, and corrosion resistance, making them suitable for use in the fabrication of implants. Demondion et al [81]. created a Ti-6Al-4V sternum following subtotal sternotomy for a single BC metastasis. The prosthetic part was a plate secured to three sides with staples, which were pulled and tightened on several ribs. During a 6-month follow-up, the patient denied having dyspnea, chest pain, or trouble going about her daily activities. A significant anterior chest wall defect was repaired in Turna et al.‘s report [82] using a titanium implant specifically created for the patient. The titanium implant was inserted after the resection of a contaminated breast tumor recurrence in a 62-year-old female patient. The tumor was in the anterior chest wall, including the sternum. Split-thickness graft and latissimus dorsi flap were also successfully employed to cover the implant. According to the report, a titanium-customized implant could be a viable alternative for patients with large chest wall tumors.

In Wen, et al. [6], the rib cage reconstruction process was carried out using a customized titanium implant fabricated through 3D printing and rapid prototyping. The mechanical characteristics of the printed implant were analysed using the ANSYS program. The results indicated that utilizing a titanium implant for reconstruction was a dependable method for addressing bony defects. Jiang et al. [7] utilized five face-centered cubic (FCC) stainless steel lattice structures as rib implants. The findings suggested that personalized implants could be created using the FCC-XYZ lattice design generated by the 125 J/mm3 laser energy density parameter. In Magesh’s research [83], titanium and UHMW were utilized as implant materials for analysis, and the results were extracted and evaluated. The findings indicated that titanium implants outperformed UHMW polyethylene implants. However, both types of implants can be utilized in chest wall reconstruction.

Despite the many advantages of using metals in the rib cage reconstruction process, they are not always suitable due to their drawbacks. The main issues with metals include their tendency to cause allergies, hypersensitivity reactions, the need for surface modifications, casting problems, incompatibility with CT and MRI imaging systems, and aesthetic concerns [10, 11]. Metals have also been associated with clinical problems like surface degradation and contamination leading to peri-implantitis [12, 13]. Therefore, it is important to explore alternative materials for metallic implants in chest wall reconstruction. Polymers are safe and scientifically certified materials currently used in biomedical engineering for orthopedic fixation and reconstruction [15–17]. A new polymeric material, PEEK (polyether ether ketone), has recently been added to the list of materials used in dentistry and orthopedics for the fabrication of implants, abutments, plates, bridges, and screws. Due to its low elastic modulus and excellent shock-absorbing ability compared to metals, this material can evenly distribute the load and dampen the stresses transmitted to the substructure parts and bone [19, 20]. Kang et al. [34] developed a novel approach for the custom design of PEEK rib prostheses that is best suited for the FDM manufacturing process. The results indicated that the centroid trajectory derived from a natural rib can provide reliable guidance for rib prosthesis design. Additionally, PEEK rib prostheses were successfully implanted and had favourable clinical outcomes.

Recently, various reinforced PEEK composites have been developed, such as carbon fiber-reinforced PEEK (CFR-PEEK) and glass fiber-reinforced PEEK (GFR-PEEK) [22–24]. In implant fabrication, these composites have gained much attention due to their biocompatibility, versatility, compatibility with modern imaging technologies, and excellent mechanical properties. In the work of Zhang et al. [31], clinical application and finite element analysis were used to assess the biomechanics of carbon fiber artificial ribs. Based on these findings, the combined simulations and clinical results validate the strong mechanical performance and biocompatibility of carbon fiber artificial ribs for chest wall reconstruction under static and dynamic loading while preserving normal respiratory function. HA-PEEK is another modification of PEEK material, which involves the addition of hydroxyapatite particles in a specific proportion [26, 27]. It is a biocompatible composite with good mechanical properties, despite its low tensile strength. This composite has been utilized as a bone substitute and scaffold in various medical specialties such as dentistry and neurosurgery, due to its osseointegration characteristics [27]. Its biocompatibility and osteoconductivity facilitate the development of osseous bridging at the interface between the implants and the surrounding tissues.

This research focuses on two primary objectives. Firstly, it conducted detailed stress and strain analyses of ribs and lungs following reconstruction with PEEK and various PEEK composite implants under two distinct loading conditions using the finite element method (FEM). Secondly, it aimed to develop sophisticated artificial intelligence models trained on material properties and FEM-derived outputs to enable precise predictions of stresses, strains, and deformations in ribs and lungs, eliminating the reliance on time-intensive simulation calculations.

Initially, the finite element model was constructed and validated with the model of a healthy rib cage constructed by Suazo et al. [43]. Five compression zones with areas of 10 cm^2^ (Z1, Z2, Z3, Z4, and Z5) simulated cardiopulmonary resuscitation techniques. The maximal von Mises stresses (MPa) and maximum compression depth (cm) were computed for validation. There are minor variations in the results (not more than 6%) when compared to the findings of Suazo et al. [43]. Following model validation, three custom-made implants were used in the process of rib reconstruction. The materials used for the implants were PEEK, hydroxyapatite PEEK 30% & 60%, carbon fiber-reinforced PEEK 30% & 60%, and glass fiber-reinforced PEEK 30% & 60%.

The von Mises stresses, tensile and compressive stresses, and strains, as well as total deformations, were extracted for the ribs cortical and cancellous bones and lung to evaluate the results. The findings demonstrated that the stresses generated on the ribs and lungs from different implants vary due to differences in their mechanical characteristics. Carbon fiber-reinforced PEEK 60% implants produced the least stresses and strains on the ribs and lungs, compared to titanium implants. However, PEEK and hydroxyapatite PEEK 30% implants produced the highest stresses and strains. Additionally, glass fiber-reinforced PEEK 30% and hydroxyapatite PEEK 60% implants distributed tensile and compressive stresses in the ribs and lungs in a similar manner.

**Fig. 16:**
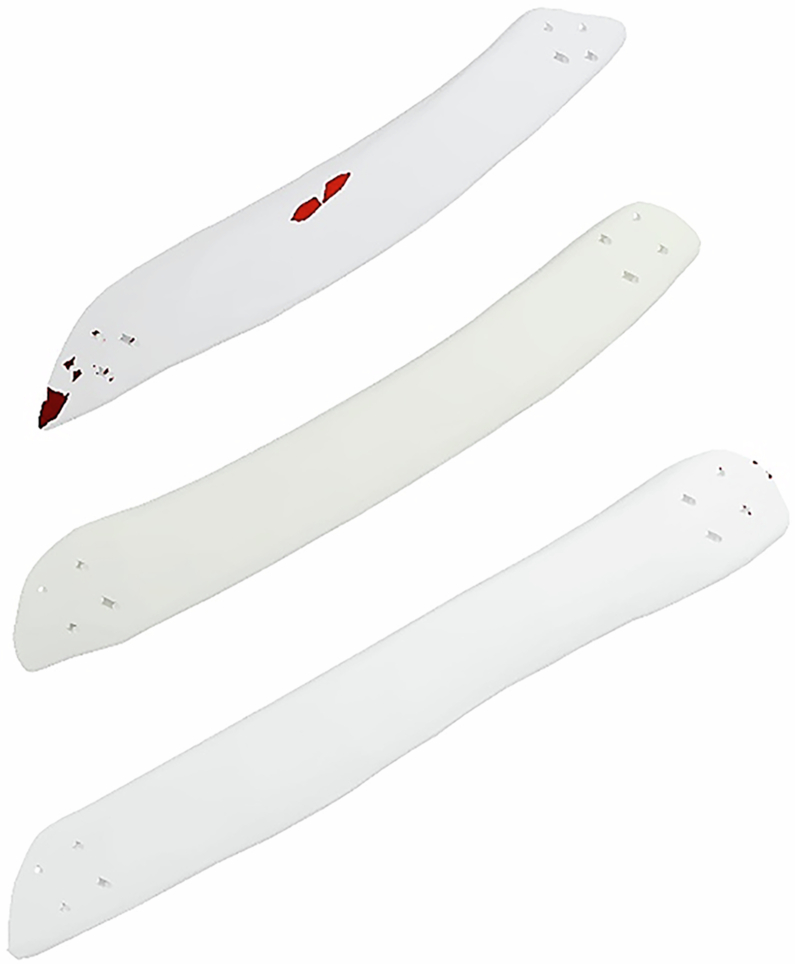
Expected fracture areas (red) in HAP 60% implants under force.1

In the failure analysis of implants, the tensile and compressive stresses were extracted (as shown in Table 4) and compared with the tensile and compressive yield strengths (Table 2) under two loading conditions. The results indicated that except for HAP 60%, all implants’ tensile and compressive stresses did not exceed their respective yield strengths. This suggests that these implants are unlikely to deform or fracture under the suggested loading conditions. However, for hydroxyapatite PEEK 60% implants, the maximum tensile stress (35.27 MPa) exceeded the tensile yield strength (32 MPa), indicating that these implants may fail under a lateral force of 1000 N (see Fig. 16).

The maximum tensile and compressive stresses for cortical and cancellous bones of ribs were calculated (Table 5 and Table 6) and compared to the corresponding tensile and compressive yield strengths (Table 2). When using titanium implants, the tensile and compressive stresses were 41.65 and 59 MPa, and 33.26 and 31.93 MPa on cortical bones under force.1 and force.2 respectively. These values increased to 70.72 and 115.8 MPa, and 42.62 and 40 MPa under force.1 and force.2 respectively, when using PEEK implants. Despite the significant increase in stresses with PEEK implants, they did not exceed the tensile and compressive yield strengths of 90 and 180 MPa respectively. Excessive strain in cortical bones can alter the microstructure of tissues and harm the interfaces between implants and ribs. Therefore, the maximum tensile and compressive strains are calculated and compared to 2500 and 4000 μԐ, respectively. Under Force 1, HAP 30% and pure PEEK implants produced tensile strains of 2,434 μԐ and 2,584 μԐ, and compressive strains of 5,658 μԐ and 6,337 μԐ, respectively. These elevated strain levels suggest that cortical bone failure is likely when using these implants.

For cancellous bone, when using titanium implants, the tensile and compressive stresses were 2.55 and 3.33 MPa under force 1, and 1.2 and 1.92 MPa under force 2, respectively. These stresses increased to 2.89 and 3.59 MPa under force.1, and 1.29 and 1.99 MPa under force.2 when using PEEK implants. However, these values did not exceed the tensile and compressive stresses of 10 and 16 MPa for cancellous bone. Additionally, the tensile and compressive strains did not exceed the critical limits of 7000 με in tension and 8000 με in compression but approached under force 1.

For the lungs, according to Rankine failure theory [62, 63], the maximum tensile and compressive stresses were extracted in Table 7 and compared to the corresponding tensile and compressive yield strengths in Table 2. When using titanium implants, the tensile and compressive stresses were 6.16 and 3.17 Pa under force.1 and force.2, respectively. These values increased to 7.4 and 7.4 Pa under force.1 and 5.8 and 5.8 Pa under force.2 when using PEEK implants. Although the stresses on the lungs were significantly higher with PEEK and hydroxyapatite PEEK 30% implants compared to titanium implants, the stress values remained within the acceptable limits of 300 Pa in tension and 800 Pa in compression. The maximum tensile and compressive strains on the lungs were 7489 and 7456 με under force.1 and 5800 and 5845 με under force.2 when using PEEK implants. These strain values were within the allowable limits of 250,000-300,000 με in tension and compression.

Finally, from the finite element analysis, it was concluded that in the ribs reconstruction process, CFP 60%, CFP 30%, GFP 60%, and GFP 30% can be used instead of titanium implants to reconstruct the defective portions of ribs to save the chest, protect the lungs, and prevent fractures. These implants are known for their superior qualities, compatibility, and lack of clinical problems. They do not induce inflammation, are safe for nearby structures, and are not prone to deformation or fracture from rapid impacts. Additionally, these composites can be formed into various shapes and have a wide range of mechanical, surface, and physical properties. Therefore, three main advantages arise from using these PEEK composites: more design freedom, decreased overall system cost, and enhanced performance [23–25]. Moreover, under the assumed conditions, hydroxyapatite PEEK 30 and 60% and PEEK implants are not recommended for use in the reconstruction process. This is because they are susceptible to damage and exert high stresses and strains on the ribs and lungs.

Combining the insights from the finite element analysis with the evaluation of regression models provides a comprehensive framework for improving rib reconstruction processes and predictive modeling in biomedical applications. The results of the regression model analysis aligned with these findings by providing predictive tools for stress, strain, and deformation in rib implants. Linear Regression and Ridge Regression models were highly efficient for real-time prediction in scenarios dominated by linear dependencies, such as stress-strain behavior under basic loading conditions.

LightGBM was optimal for applications requiring precision and the ability to model both linear and non-linear relationships, such as designing rib implants with complex mechanical properties. Neural Networks were ideal for exploratory or simulation-heavy analyses due to their ability to capture intricate non-linear patterns but are less practical for real-time applications due to their computational demands. SVR, while capable of modeling non-linear dependencies, was less suitable for rapid analysis due to higher error rates and longer processing times. Finally, Decision Trees, with their focus on interpretability, were more suited for exploratory analyses of hierarchical material and loading influences.

By integrating finite element analysis findings with the predictive capabilities of these regression models, researchers can better tailor rib implant designs to specific mechanical and clinical requirements. This dual approach not only optimizes implant performance but also reduces the need for extensive computational simulations, enabling faster and more accurate predictions in real-world biomedical applications. Together, these methodologies form a holistic strategy for improving patient outcomes and advancing the field of rib reconstruction.

The current study has certain limitations. Firstly, all biological and implant materials were modeled as linear elastic, isotropic, and homogeneous, consistent with previous thoracic finite element analyses. Although this represents a simplification—particularly for soft tissues such as the lungs—it was adopted to ensure computational stability and enable direct comparison between implant materials under uniform boundary conditions. Accordingly, the findings are valid within the assumptions of linear elasticity and small deformations.

Secondly, while impact loading was represented through equivalent static forces to simplify the computational process, this approach neglects inertial effects, strain-rate sensitivity, and dynamic energy dissipation mechanisms. As a result, the obtained results reflect the quasi-static mechanical response of the rib–implant assembly rather than its full dynamic behavior. Future work should employ explicit dynamic solvers to accurately capture transient impact phenomena and further validate the implants’ mechanical performance under real-world loading conditions.

## Conclusion

The aim of this research was twofold: first, to evaluate the feasibility of using PEEK and PEEK composites for rib reconstruction through finite element analysis (FEM); and second, to develop artificial intelligence models capable of predicting the stresses, strains, and deformations of the ribs and lungs.

Based on the results, and within the limitations of the current study, the following conclusions were drawn:

1. Carbon fiber-reinforced PEEK 60% implants produced the least stresses and strains on the ribs and lungs.
2. PEEK and hydroxyapatite PEEK 30% implants produced the highest stresses and strains on the ribs and lungs and might damage the surrounding ribs.
3. The stresses distributed in the ribs and lungs using carbon fiber-reinforced PEEK 30 and 60% implants were nearly the same as those in titanium implants.
4. Glass fiber-reinforced PEEK 30% and hydroxyapatite PEEK60% implants distributed the tensile and compressive stresses in the ribs and lungs in a nearly similar manner.
5. Hydroxyapatite PEEK 60% implants were susceptible to fracture because the generated tensile stresses were greater than their tensile strength, under an impact force of 1000 N.
6. By using all implants, the tensile and compressive stresses and strains on the lungs were within the allowable limits.
7. The integration of finite element analyses with artificial intelligence (AI)-based regression models significantly optimized the evaluation process.
8. Regression models, particularly Linear and Ridge Regression, achieved near-perfect predictive accuracy (R² = 1.0), with exceptional computational efficiency, making them ideal for real-time material assessments.
9. Advanced models like LightGBM further enhanced non-linear predictive accuracy while maintaining computational efficiency.

## Data Availability

The data that support the findings of this study are available from the corresponding author upon reasonable request.

## Abbreviations

PEEK: Poly ether ether ketone
CFR-PEEK (CFP): Carbon Fiber Reinforced PEEK
GFR-PEEK (GFP): Glass Fiber Reinforced PEEK
HA-PEEK: Hydroxyapatite PEEK
FEM: Finite element model
AI: Artificial Intelligence
RBF: Radial Basis Function
FCN: Fully Connected Neural Networks
DL: Deep Learning
ML: Machine Learning
MSE: Mean Squared Error
RMSE: Root Mean Squared Error
MAE: Mean Absolute Error
